# An Experimental Study of a Micro-Projection Enabled Optical Terminal for Short-Range Bidirectional Multi-Wavelength Visible Light Communications

**DOI:** 10.3390/s18040983

**Published:** 2018-03-26

**Authors:** Hsi-Hsir Chou, Cheng-Yu Tsai, Jhih-Shan Jiang

**Affiliations:** Department of Electronic and Computer Engineering, National Taiwan University of Science and Technology, Taipei 10607, Taiwan; m10302338@mail.ntust.edu.tw (C.-Y.T.); m10302330@mail.ntust.edu.tw (J.-S.J.)

**Keywords:** visible light communications, spatial light modulator, liquid crystal on silicon device

## Abstract

A micro-projection enabled short-range communication (SRC) approach using red-, green- and blue-based light-emitting diodes (RGB-LEDs) has experimentally demonstrated recently that micro-projection and high-speed data transmission can be performed simultaneously. In this research, a reconfigurable design of a polarization modulated image system based on the use of a Liquid Crystal on Silicon based Spatial Light Modulator (LCoS-based SLM) serving as a portable optical terminal capable of micro-projection and bidirectional multi-wavelength communications is proposed and experimentally demonstrated. For the proof of concept, the system performance was evaluated through a bidirectional communication link at a transmission distance over 0.65 m. In order to make the proposed communication system architecture compatible with the data modulation format of future possible wireless communication system, baseband modulation scheme, i.e., Non-Return-to-Zero On-Off-Keying (NRZ_OOK), M-ary Phase Shift Keying (M-PSK) and M-ary Quadrature Amplitude Modulation (M-QAM) were used to investigate the system transmission performance. The experimental results shown that an acceptable BER (satisfying the limitation of Forward Error Correction, FEC standard) and crosstalk can all be achieved in the bidirectional multi-wavelength communication scenario.

## 1. Introduction

With the increasing popularity of smartphone-based portable communication devices (PCDs) in the modern networked community, optical terminals based on these PCDs for applications in optical wireless communication technologies have received much attention since in the near future, over 4~5 billion smartphones and 2 billion active tablets are expected to access Internet service [1] either through wireless link at microwave frequencies or optical wireless link. In particular, the number of machine to machine (M2M) or device to device (D2D) connections in mobile networks is also projected to surpass 15 billion and 26 billion in 2018 and 2022 respectively [2]. However, the data transmission performance provided from those short-range communication (SRC) technologies such as Bluetooth [3], ZigBee [4] and Near Field Communication (NFC) [5], which might have already existed commercially on current PCD is still limited to less than five Mb/s [3,4,5].

Although recent developed visible light communication (VLC) technologies [6] are promising to offer a higher data transmission rate for PCD application, a well solution to integrate VLC into a PCD to meet the market requirement remains a big challenge [7]. Moreover, it is also unclear how to use PCD as an optical terminal to receive optical signal efficiently. Although using a CMOS (Complementary Metal-Oxide Semiconductor) based optical camera module on PCD to serve as an optical terminal for the establishment of an optical wireless communication link has been proposed [8], recent performance presented have shown that only fewer Kb/s can be achieved [9].

In contrast to using a CMOS optical camera as an optical terminal, an optical terminal based on micro-projection architecture of PCD seems more attractive, since it is widely expected that the micro-projector integrated with VLC technology will be a vital part of a new generation PCD [7]. In particular, a micro-projection enabled SRC system based on VLC technology providing a nearly gigabit data transmission rate has experimentally demonstrated and proposed to serve as an alternative communication approach for PCD application [10].

However, the work that was presented in [10] was based on one-way transmission, and multi-wavelengths were not transmitted simultaneously. This encouraged the development of an optical terminal based on micro-projection architecture of PCD to establish a bidirectional optical wireless communication link for M2M or D2D applications. In this research, optical terminal based on micro-projection architecture of PCD for bidirectional multi-wavelength visible light communication application is proposed and experimentally demonstrated.

## 2. System Design and Experimental Setup

**Figure 1:**
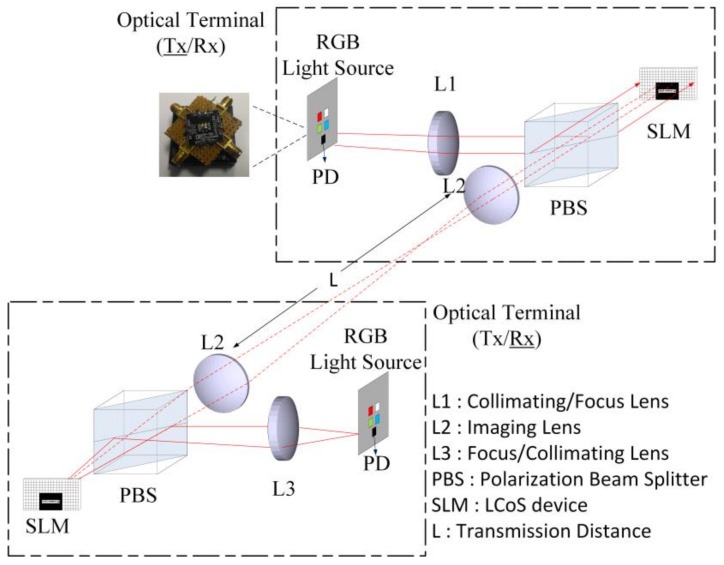
Architecture of short-range bidirectional visible light communication (VLC) based on optical terminals (OTs).

The scenario of portable optical terminal (OT) based on micro-projection architecture for short-range bidirectional multi-wavelength visible light communications is illustrated as in Figure 1. Each OT is based on an LCoS-based micro-projection architecture [10], for which it has been demonstrated that data transmission is possible while performing micro-projection simultaneously. The physical layer components of each OT are identical. It includes a RGB-LEDs (red-, green- and blue-based light-emitting diodes) light source manufactured from Philip (Luxeon Z series), a PD (photo-detector) for signal detection, a lens, L1/L3 with a diameter of 50 mm for collimating/focusing, a 50:50 PBS (polarization beam splitter) for polarization beam splitting, a LCoS-based SLM (Liquid Crystal on Silicon based Spatial Light Modulator) and an image lens, L2 with a diameter of 50 mm. All the lens components and PBS were manufactured from Thorlabs. The advantages of using RGB-based LEDs as the light source of an OT are that a higher modulation bandwidth as well as multiple wavelengths can be used compared with the conventional light source, i.e., a blue LED based white light source, in which the available modulation bandwidth is inherently restricted by the phosphor. However, the main challenge for short-range bidirectional multi-wavelength visible light communications based on OTs is that multiple wavelengths received by the OT should be selected dynamically without the need to enforce additional hardware modifications, i.e., employment of more PDs. In this research, the experimental work of using OT as transmitter (OT_Tx) and receiver (OT_Rx) for bidirectional multi-wavelength visible light communications that have investigated is presented and demonstrated.

In our experiments, the use of the OT in the transmitter mode (OT_Tx) is similar to the work presented previously [10]. As illustrated in Figure 1, a Philip Luxeon Z series RGB-based LEDs [11], which is a low cost component and available commercially, was used as the light source of the OT_Tx. Three LED chips, which have the central wavelengths of 650 nm, 550 nm and 450 nm respectively, are included within this light source. The 3-dB modulation bandwidths of each LED chip within the OT architecture were measured to be 8 MHz, 16 MHz and 12 MHz for 650 nm, 550 nm and 450 nm LED chips respectively. These LED chips have also mounted on a MCPCB (metal core printed circuit board, 20 mm × 20 mm) for data transmission (OT_Tx). The data for OT_Tx transmission were generated randomly and modulated offline by a computer software (Matlab) from a PC (Personal Computer). These signals were transferred to the memory of an AWG (arbitrary waveform generator, Keysight 33621A) which has a sampling rate of 1 GSa/s. The emitted lights of these LED chips were driven individually by signals that have been modulated offline from AWG and biased separately by DC currents through low-frequency Bias Tees. The DC current used was fixed to 350 mA since it has a better performance observed from our research works.

The operation principle of the OT in the forward direction link is that multiple wavelengths (i.e., red, green and blue wavelengths) transmitted from light sources are collimated by the collimating lens, L1 first and are then selected by a PBS before incident on the LCoS-based SLM. Since the LCoS-based SLM is sensitive to the polarization property of the incident light, only one polarization state of the incident light which was selected by a PBS in advance can be modulated. Although this polarization sensitivity problem will result a higher system light loss, a typical polarization conversion system (PCS) [12,13] can be used to compensate this loss. The LCoS-based SLM device used was a phase only microdisplay (JD 9554, JDC, Hsinchu, Taiwan) and all pixels on the device can be controlled through the upload of a hologram pattern that has designed. These patterns can be binary, multilevel or continuous. In our experimental setup, the LCoS-based SLM was sectioned into a different area in which each area has a sub-hologram uploaded. The majority of the sub-holograms on the LCoS device were used to perform the polarization modulation of the incident light, which reflects the light into free-space through the image lens (L2) except that only one sub-hologram corresponding to a SVGA (super video graphics array) resolution (800 × 600 pixel) was used for micro-projection application in our proof of concept research.

**Figure 2:**
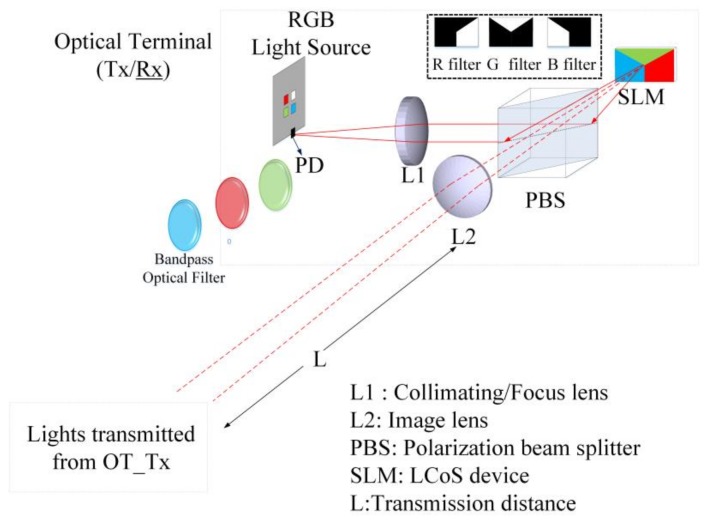
Design of OT_Rx for reverse directional link.

**Figure 3:**
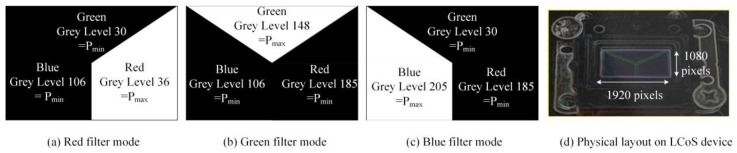
Design of LCoS-based SLM as tunable filter.

**Figure 4:**
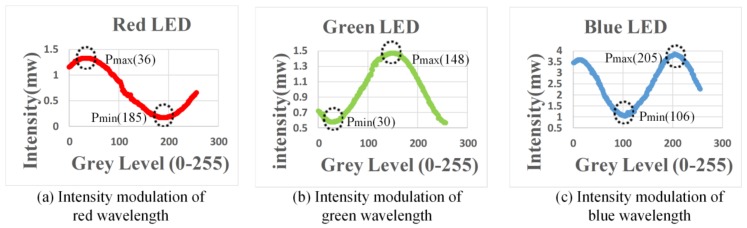
Calibration results of LCoS-based SLM using RGB-LEDs.

When multiple wavelengths of lights were transmitted from OT_Tx, another OT (OT_Rx) in the reverse direction link can be used to receive these lights for communications. However, in order to select the wavelength accordingly, the OT_Rx should be operated as a dynamic bandpass optical filter given that only one PD is used. For OT_Rx to be operated as a dynamic bandpass optical filter and select wavelength accordingly, the LCoS-based SLM can be collaborated with or without physical bandpass optical filters, as illustrated in Figure 2. In the configuration of an LCoS-based SLM without physical bandpass optical filters, the LCoS-based SLM is operated as a tunable filter to reflect a desired wavelength into the PD accordingly. In order to reflect or block the wavelengths of 650 nm, 550 nm and 450 nm from red-, green- and blue- LED chips respectively, the LCoS-based SLM as illustrated in Figure 3, was divided into three section areas, which are “Red”, “Green” and “Blue”. Each section area was responsible for each corresponding wavelength separately. As shown in Figure 3a, when the LCoS-based SLM is in Red filter mode in which the red wavelength is a desired one to pass, the section area of “Red” on LCoS device was setup to have the maximum reflection power for red wavelength. The other section areas on LCoS device were setup to have the minimum power reflection for green and blue wavelengths respectively. The design of the LCoS-based SLM to modulate the reflected intensity of 650 nm, 550 nm and 450 nm wavelengths respectively was based on our calibration results in terms of using RGB-LEDs light source as shown in Figure 4.

Although in each filter mode, the LCoS-based SLM can be setup to have the best reflection efficiency for each corresponding wavelength, the crosstalk resulting from the light of unwanted wavelengths is still unavoidable. Therefore, in order to improve the system crosstalk, physical bandpass optical filters were in turn used in front of the PD to further investigate the property of the crosstalk.

At the receiver part, a focusing lens L3 was setup in front of a PD in order to improve the collection efficiency of the incident light transmitted from free-space. The PD (PDA 10A, Thorlabs) used was a Si free-space amplified photodetector which has an active area of 0.8 mm^2^ [14] and a responsivity of 0.385, 0.25 and 0.175 amperes per watt (A/W) for red, green and blue wavelengths respectively. For the multi-wavelength communication scenario, several PDs, which were devoted to each corresponding wavelength within the receiver, should be used and physical bandpass optical filters are also essential components that are normally used in front of each detector in order to choose a desired wavelength and cut off the other unnecessary wavelengths. Although this kind of configuration offers the possibility for the terminal users to receive multi-wavelength simultaneously, it is not so practical for a terminal device to accommodate these PDs within a limited physical space. Moreover, the effectiveness resulted from the physical bandpass optical filters in the proposed optical terminal architecture has also not evaluated properly. In our experimental research works, by simplifying the conventional approach used in [15], the physical bandpass optical filter corresponding to each dedicated wavelength was in turn used in front of a PD to investigate the data transmission performance of bidirectional communications based on the proposed OT architecture. Although the optical filters that were used have the central wavelength of 450 nm, 550 nm and 650 nm respectively (Thorlabs, FB450-40, FB550-40 and FB650-40), their transmittances are not identical at each corresponding wavelength and therefore a varied degradation of system performance resulted. An amplifier with a gain of 11dB (Minicircuits) was used to amplify the received signals from the PD again before these signals were recorded by an oscilloscope which has a real-time sampling rate of 5 GSa/s (Keysight DSOX 4104A). A VSA (vector signal analysis) software (Keysight VSA89600) was then used directly to analyze these recorded signals which have not processed with any signal processing technique, i.e., equalization from the real-time oscilloscope.

## 3. Results and Discussions

A bidirectional multi-wavelength visible light communication link based on micro-projection enabled OTs at a transmission distance over 0.65 m were initially established to evaluate the system performance in our proof of concept experiments. Although the experimental works were conducted based on a half-duplex communication scenario, the proposed communication approach based on micro-projection enabled OTs can be applied to a full-duplex communication scenario through an appropriate design of LCoS-based SLM since each OT has identical physical components. For the proposed communication system architecture to be compatible with the data modulation format of future possible wireless communication system, several baseband modulation schemes were initially used in our experimental research works to evaluate the proposed system in terms of the highest possible data transmission rate at a BER (Bit Error Rate) of less than 10^−3^. Since several multicarrier modulation schemes, i.e., OFDM and DMT which have been widely used to enhance the bandwidth efficiency of optical wireless communications are all based on the employment of the baseband modulation schemes, i.e., M-ary PSK (Phase Shift Keying) and M-ary QAM (Quadrature Amplitude Modulation) [16], in our experimental research works, NRZ_OOK (Non-Return-to-Zero On-Off-Keying), M-ary PSK and M-ary QAM were therefore used to compare the data transmission performance. The measured eye diagrams and constellation diagrams from the oscilloscope and VSA software respectively were used to estimate and calculate the BERs of the system in the performance evaluation of these baseband modulation schemes used in our experiments. The experimental results in terms of crosstalk and data transmission performance are reported and discussed as follows: 

### 3.1. Analysis of Crosstalk

**Figure 5:**
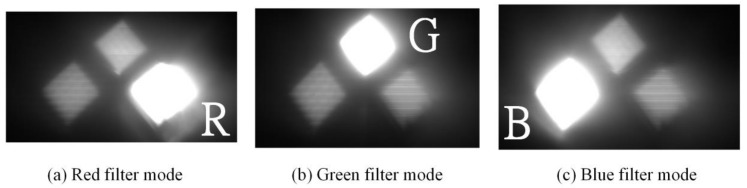
Received lights at OT_Rx without adding physical bandpass optical filters.

**Figure 6:**
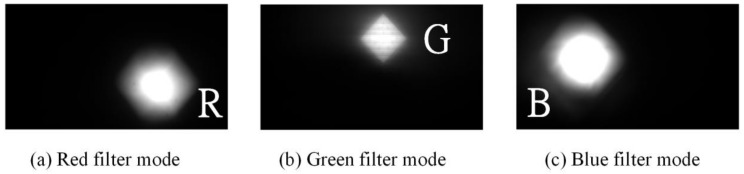
Received lights at OT_Rx with the aid of physical bandpass optical filters.

In our experimental works, multi-wavelengths were transmitted simultaneously from OT_Tx, and therefore, another OT (OT_Rx) in the reverse direction was used to receive and operate as a tunable bandpass optical filter to select wavelength accordingly. In order to study the feasibility of using OT_Rx to be operated as tunable bandpass optical filter, the LCoS-based SLM was collaborated with and without the aid of physical bandpass optical filters in our experiments. The crosstalk of the system, $C_{T}$ received at the OT_Rx was due to the fact that the other unwanted wavelengths, which should have been cut off by LCoS-based SLM, were received. Therefore, the system crosstalk was defined by the ratio of the received optical power at PD from unwanted wavelengths, which should have been cut off by the LCoS-based SLM, to the received optical power at the same PD from a selected wavelength, which was chosen to be reflected from the LCoS-based SLM [17].

$$C_{T}=10\log_{10}\frac{P_{n}}{P_{t}}$$

where $P_{n}$ is the optical power of unwanted wavelengths, which should have cut off, and $P_{t}$ is the optical power of a selected wavelength. When the LCoS-based SLM was used as a tunable bandpass optical filter to select wavelength accordingly without adding physical bandpass optical filters in front of the PD, the received light intensity of each wavelength at the PD were measured and the calculated results shown that the crosstalk is at a range of −3.8 dB~−4.8 dB. The captured images of the received light through CCD (Charge-coupled Device) camera as illustrated in Figure 5 show that the unwanted wavelengths were not completely blocked by LCoS-based SLM and therefore physical bandpass optical filters were used to improve the crosstalk. The measurement results show that with the aid of physical bandpass optical filters placed in front of the PD, the crosstalk has been dramatically improved to the range of −10.4 dB~−12.1 dB. The captured images of the received light through CCD camera as illustrated in Figure 6 have shown that the unwanted wavelengths were completely blocked. Therefore, in the following data transmission performance evaluations, physical bandpass optical filters were in turn used in front of the PD at OT_Rx.

### 3.2. Data Transmission Test

**Figure 7:**
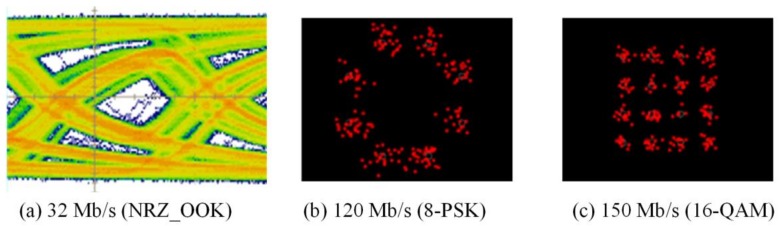
Micro-projection and data transmission of Red LED with bandpass filter (BER = 10^−3^).

**Figure 8:**
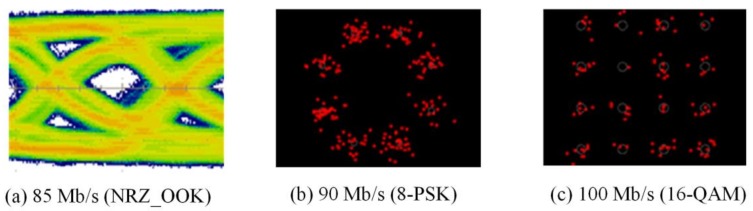
Micro-projection and data transmission of Green LED with bandpass filter (BER = 10^−3^).

**Figure 9:**
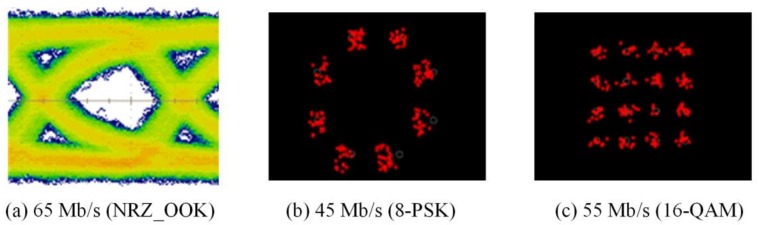
Micro-projection and data transmission of Blue LED with bandpass filter (BER = 10^−3^).

**Figure 10:**
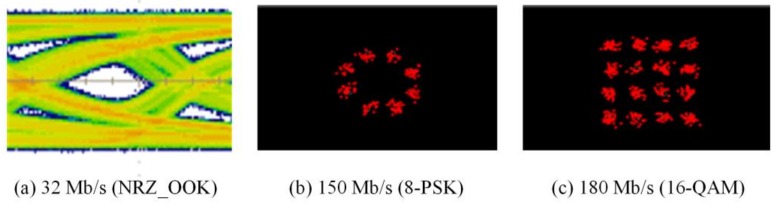
Pure data transmission rate of Red LED with bandpass filter (BER = 10^−3^).

**Figure 11:**
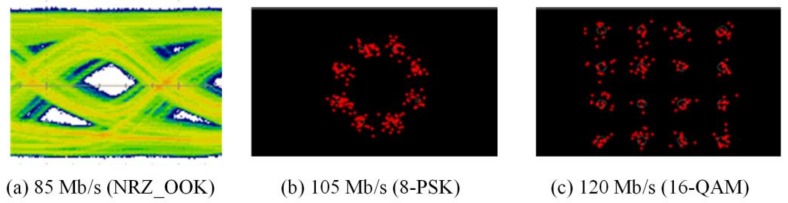
Pure data transmission rate of Green LED with bandpass filter (BER = 10^−3^).

**Figure 12:**
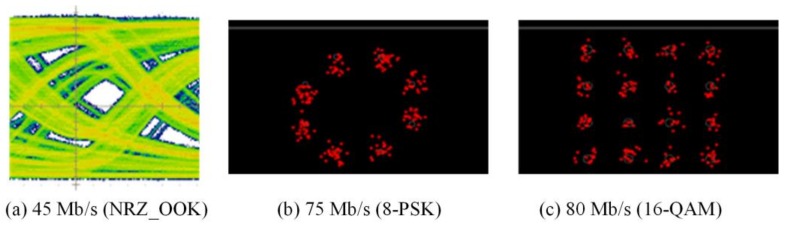
Pure data transmission rate of Blue LED with bandpass filter (BER = 10^−3^).

The experimental evaluations of OTs for bidirectional multi-wavelength communication were conducted by two scenarios, in which the OT_Tx was used to perform multi-wavelength transmissions either with or without micro-projection function simultaneously. In order to receive multi-wavelength transmitted from free-space, physical bandpass optical filters were used in the configuration of the receiver part. When multi-wavelengths were transmitted, while micro-projection function was also performed, the measured results of red, green and blue wavelengths as illustrated in Figure 7, Figure 8 and Figure 9, demonstrated that the maximum data transmission rate at a BER of $10^{-3}$ composed by these three wavelengths using the modulation scheme of NRZ_OOK, 8-PSK and 16-QAM are 182 Mb/s, 255 Mb/s and 305 Mb/s respectively. When multi-wavelength were transmitted, while micro-projection function was not performed, the measured results of red, green and blue wavelengths as illustrated in Figure 10, Figure 11 and Figure 12, shown that the maximum data transmission rate at a BER of $10^{-3}$, composed by these three wavelengths using the modulation scheme of NRZ_OOK, 8-PSK and 16-QAM are 162 Mb/s, 330 Mb/s and 380 Mb/s respectively. 

From the comparisons of the measurement results, it is clearly that the data transmission performance of all the modulation schemes except the amplitude modulation scheme, i.e., NRZ_OOK, has improved when data were transmitted while micro-projection was not performed. The major problem that the amplitude modulation scheme, i.e., NRZ_OOK was not improved when no image data was presented was resulted from the low contrast ratio (CR) of the LCoS device when using RGB-LEDs. Since NRZ_OOK is a pulse amplitude modulation, a low CR of LCoS device will not be able to perform intensity/amplitude modulation efficiently. The data rate improvement of NRZ_OOK, are therefore not absolutely positive. The low CR of the LCoS device resulted from the fact that the RGB-LEDs light source used is a non-coherent light source and therefore the problem of low CR can be improved while coherent light sources, i.e., laser diodes are used.

From the comparisons of the measurement results, which all satisfy the Forward Error Correction (FEC) limitations [18], the 16-QAM can achieve the highest data transmission rate either when micro-projection was performed with data transmission simultaneously or not. However, when the micro-projection was performed simultaneously with data transmission, it is obvious that the performance degradation in terms of a lower data transmission rate has resulted. Nevertheless, the proposed OT based on micro-projection architecture for bidirectional multi-wavelength VLC still offers a better data transmission performance than using CMOS optical camera-based VLC.

## 4. Conclusions

In this experimental research, we have reported and demonstrated a short-range bidirectional multi-wavelength VLC system based on micro-projection enabled optical terminals. To the best of our knowledge, this is the first time that a micro-projection enabled optical terminal capable of bidirectional multi-wavelength visible light communication has been presented. In order to make the proposed communication system architecture compatible with the data modulation format of future wireless communication system, several baseband modulation schemes, i.e., NRZ_OOK, M-ary PSK and M-ary QAM, which have widely employed by a multicarrier modulation scheme to raise the bandwidth efficiency, were used to investigate the system transmission performance. The measurement results show that the maximum data transmission rate of 380 Mb/s (BER = 10^−3^) can be achieved based on a pure bidirectional multi-wavelength communication scenario. The analysis of the system performance also indicated that physical bandpass optical filter can be used to improve the system crosstalk. Compared with the optical terminal using CMOS optical camera as the receiver for bidirectional VLC, the proposed optical terminal provides a better approach for direct implementation of a bidirectional VLC system and offers a higher data transmission performance.
